# Light-emitting diodes with surface gallium nitride *p–n* homojunction structure formed by selective area regrowth

**DOI:** 10.1038/s41598-019-40095-7

**Published:** 2019-03-01

**Authors:** Ming-Lun Lee, Shih-Sian Wang, Yu-Hsiang Yeh, Po-Hsun Liao, Jinn-Kong Sheu

**Affiliations:** 1 Department of Electro-Optical Engineering, Southern Taiwan University of Science and Technology, Tainan, 71005 Taiwan; 2 Department of Photonics and Advanced Optoelectronic Technology Center, National Cheng Kung University, Tainan City, 70101 Taiwan

## Abstract

In this study, the blue light-emitting diode (LED) structures based on gallium nitride (GaN) were presented. Each structure possessed a surface GaN *p–n* junction, which was formed through selective area regrowth on an InGaN/GaN multiple quantum well (MQW) structure and served as the carrier injector. The LEDs that showed efficient hole injection and current spreading were configured to form a *p*-type GaN layer between the MQW and regrown *n*-type GaN top layer. These LEDs exhibited higher luminous efficiency and lower operation voltage than the LEDs with regrown *p*-type GaN top layers. The LEDs with *n*-type GaN top layers emitted single-peak spectra at approximately 450 nm under a forward bias. The UV peak at 365 nm (i.e., the GaN band-edge emission) was absent because the regrown surface GaN *p–n* junctions behaved as carrier injectors rather than photon injectors. In other words, the single-peak blue emission was not generated by the optical pumping of UV light emitted from the surface *p–n* GaN homojunction.

## Introduction

White light-emitting diodes (WLEDs) are next-generation semiconductor light sources that can be used for addressing energy shortage and environmental issues^1^. Commercial WLEDs are mainly produced by combining gallium nitride (GaN)-based blue LED and yellow phosphor or combining red, green, and blue LEDs. Highly efficient green or blue LEDs made of InGaN-based semiconductors are considered key devices regardless of the selected method. The external quantum efficiency (EQE) of an LED depends on the internal quantum efficiency (IQE) and light extraction efficiency (LEE) of photons emitted from the active layer of the LED. Most studies aimed at enhancing the LEE focused on reducing photon loss due to total internal reflection inside LEDs^2–5^. At present, efficiency droop, which is the nonthermal rollover of IQE at a high-current density in III-nitride LEDs remains a critical issue. Several proposed mechanisms, such as Auger recombination in the active region and carrier leakage from the active region, have been attributed to be the origin of efficiency droop^6^. Regardless of the main origin of efficiency droop, current density (i.e., carrier density) in the active region dominates the onset of these mechanisms. Uniform carrier distribution throughout the active layer (i.e., InGaN/GaN-based quantum wells [QW]) in the vertical direction perpendicular to the heteroepitaxial layers has been considered a key issue for alleviating efficiency droop in nitride-based LEDs^6^. Current spreading in the horizontal direction parallel to the heteroepitaxial layers has been considered a critical issue as well because current crowding around the electrodes of LEDs causes high carrier density and ultimately results in efficiency droop^7,8^.

Recently, Kivisaari *et al*.^9–11^ proposed GaN-based LEDs with current injection structures in which carriers from *p–n* junctions diffuse to active regions outside *p–n* junctions. They indicated that the diffusion-assisted carrier injection (DACI) provides a possible solution for alleviating efficiency droop in III-nitride LEDs. In contrast to conventional GaN-based p-i-n LEDs, the proposed LEDs, in which active regions are under *p–n* junctions, provide a DACI mechanism that enables electron and hole diffusion laterally to active regions and thereby may lead to an efficient current spreading over a large area in an LED and reduce the efficiency droop effect. In this study, LED structures with InGaN/GaN multi-quantum well (MQW) active layers underneath GaN *p–n* junctions formed by selective area regrowth technique are experimentally demonstrated. The device area before the regrowth process of an *n*-type GaN (or p-GaN) top layer by metal-organic vapor-phase epitaxy (MOVPE) is defined by patterning a silicon dioxide (SiO_2_) mask layer on an InGaN/GaN MQW structure with *p*-GaN (or *n*-GaN) cap layer. During device processing, dry etching is not required for the exposure of *p*-GaN (or *n*-GaN) layers for the formation of *p*-type (or *n*-type) ohmic contacts^12^. A conventional GaN-based p-i-n LED structure with an InGaN/GaN MQW active region sandwiched between *p*- and *n*-type GaN regions is also prepared for comparison (Figure. S1, Supplementary Information). The detailed processing procedures and related results, such as the electrical and optical properties of fabricated LEDs, are then discussed.

## Methods

**Figure 1:**
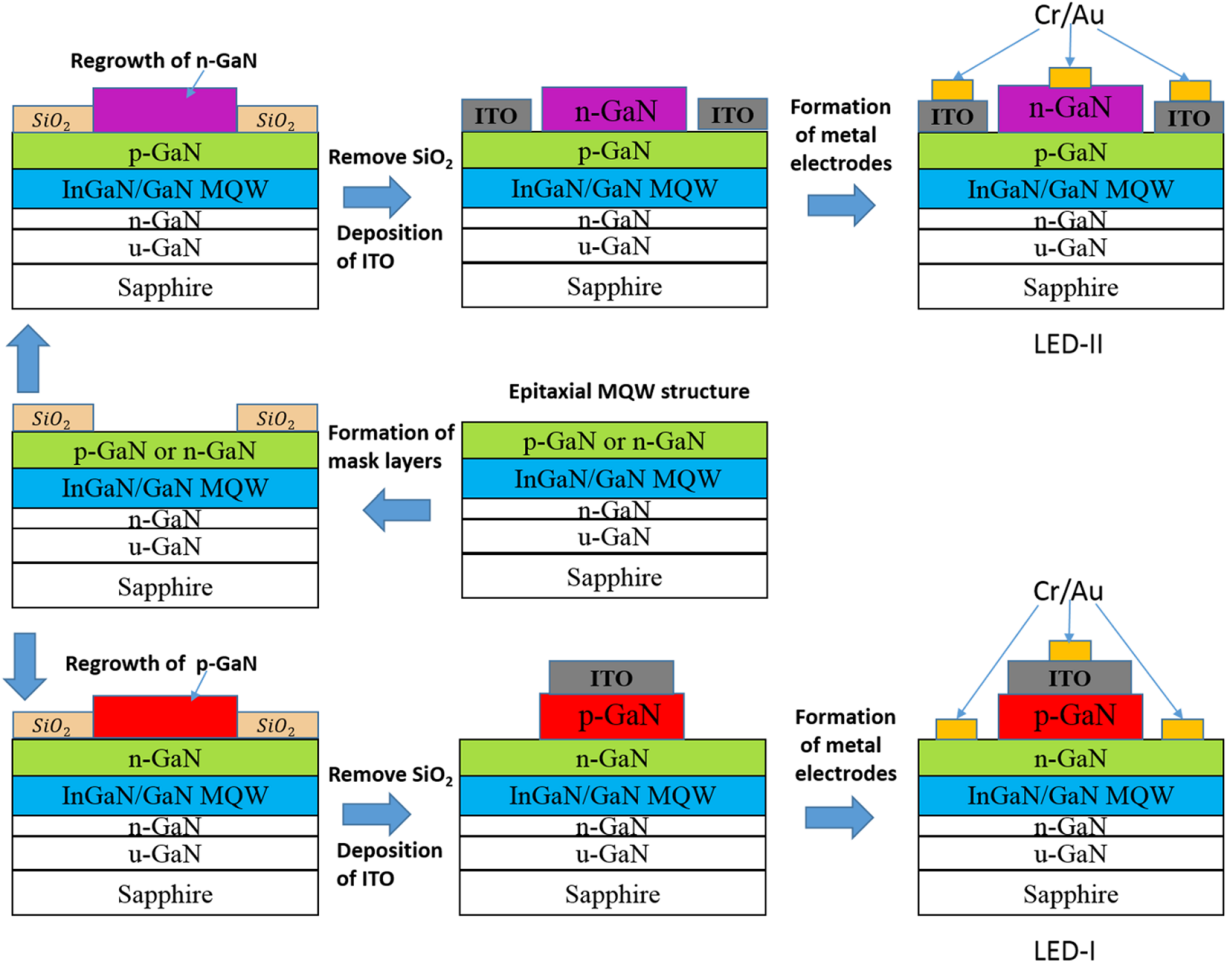
Layer structure of InGaN/GaN MQW structures and fabrication steps for the experimental devices (LED-I and LED-II).

The regrowth templates used in this study, including a 30 nm-thick GaN nucleation layer grown at 530 °C, a 3 μm-thick undoped GaN layer grown at 1000 °C, a 10-pair In_0.23_Ga_0.77_N/GaN MQW grown at 750 °C, and an 80 nm-thick Mg-doped *p*-GaN cap layer grown at 950 °C, were sequentially grown on sapphire substrates in a vertical metalorganic vapor-phase epitaxy (MOVPE) reactor (Emcore D-180). For the MQW, each pair of QW consisted of a 3 nm-thick In_0.2_Ga_0.8_N well layer and 12 nm-thick GaN barrier layer. An 80 nm-thick Si-doped *n*-GaN cap layer rather than a *p*-GaN cap layer on the MQW was prepared and used as the template for the subsequent regrowth process. The detailed parameters of each layer, including thickness and doping concentration, were described in the Supplementary Information. Figure 1 depicts the layer structure of the InGaN/GaN MQW structures.

A 1 μm-thick SiO_2_ layer was deposited by plasma-enhanced chemical vapor deposition (PD-100ST PECVD, Samco Inc.) on the MQW templates. A 2 µm-thick photoresistor (PR, AZ 5214) layer was selectively formed on the SiO_2_ layer through photolithography and PR developer (tetramethyl ammonium hydroxide: H_2_O = 97.62: 2.38%). A series of openings on the SiO_2_ layer were formed to serve as the mask layer for the subsequent selective area regrowth of *n*-GaN (or *p*-GaN) on the *p*-GaN (or *n*-GaN) top layer of the MQW structures. The SiO_2_ layer was selectively etched away with a buffer oxide etchant (HF: NH_4_F = 14.35: 85.7%) and then cleaned with deionized water and isopropyl alcohol. Figure 1 also displays the fabrication steps for the experimental devices used after the regrowth of *n*-GaN or *p*-GaN top layers on the MQW templates. The regrown 200 nm-thick *p*-GaN in LED-I presented typical hole concentrations of approximately 3 × 10^17^/cm^3^. Dry etching on GaN-based layers was not required for the formation of the mesa structures of *p–n* junctions in the experimental LEDs because the *n*-GaN layers were exposed only when the SiO_2_ mask layers were removed with a wet etching solution (buffer oxide etchant) after selective area regrowth. The LEDs fabricated through the same processing procedures for regrowing *n*-GaN on the *p*-GaN topmost layers of MQW structures were also prepared for comparison and labeled as LED-II, as shown in Fig. 1. Furthermore, *p*-type ohmic contact in LED-I was formed by depositing a 200 nm-thick indium tin oxide (ITO) film prepared by radio frequency sputtering on the *p*-GaN area. A Cr/Au (50/1,000 nm) bilayer metal was deposited on the ITO and *n*-GaN area for the formation of anode and cathode electrodes, respectively^13^.

The dimension of the fabricated devices was 370 × 360 µm^2^ (See Figures S2 and S3, Supplementary Information, for the detailed device configuration). The reason that this dimension was used was unspecified. However, we anticipated that carrier transport may vary with device dimension. For device characterization, current–voltage (*I–V*) characteristics were measured at room temperature by using a semiconductor parameter analyzer (HP4156C). The light output power–current (L–I) characteristics of each LED were determined with a calibrated integrating sphere. The electroluminescence (EL) spectra of the LEDs were obtained with a calibrated charge-coupled device camera mounted on a microscope with a current source meter (Keithly 2400).

## Results and Discussion

**Figure 2:**
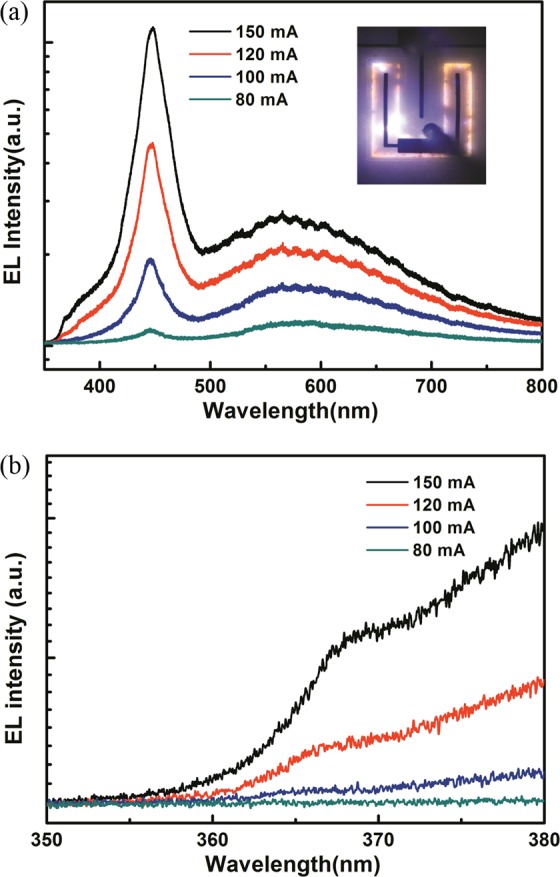
(**a**) Typical current-dependent EL spectra obtained from LED-I (**b**) display range is set between 350 nm and 380 nm to highlight the UV region of EL spectra obtained from LED-I. The inset of (**a**) shows the emission image of LED-I driven at a current of 100 mA.

Figure 2(a) displays the current-dependent EL spectra obtained from LED-I. The emission peak at approximately 450 nm was observed, indicating that the injected carriers from the surface GaN *p–n* homojunction diffused to the buried InGaN/GaN QWs and recombined to yield a blue emission. The yellow luminescence (YL) band, a well-known unique emission of GaN, was observed as well^14,15^. Kivisaari *et al*.^9^ attributed the emitted YL band from the diffusion-assisted LED to the radiative recombination involving defect states in the GaN layers. The YL emission was attributed to the recombination of a fraction of injected carriers with native defects in their GaN homojunctions especially at a low excitation (i.e., low injection current). The presence of the YL band in the EL spectra was attributed to the low injection efficiency of hole diffusion to the InGaN/GaN QWs. A reliable EL spectrum was not obtained in LED-I when the driving current was lower than 80 mA because most of the injected carriers (i.e., injection current) laterally moved within the surface *p–n* homojunctions rather than diffusing into the buried QWs. In other words, most of the injected carriers, especially for the holes, were confined in the surface *p–n* homojunction even when the injection current reached a high value of 80 mA. In principle, the decrease in potential barrier height promotes the carriers diffusing into QWs. The potential barrier, which is at the interface between the QWs and the GaN *p–n* homojunction, decreases with the increase of applied voltage (i.e., injection current). The injected holes recombined with the injected electrons in the QWs instead of recombining with defect states in the *n*-GaN layer. However, in LED-I, the YL band did not disappear even when the current injection increased to 150 mA. This phenomenon was attributed to the fact that the holes passed through the *n*-GaN layer before recombining with the electrons in QWs. Consequently, a part of the injected electrons and holes recombined with each other or with the native defects in the surface *p–n* homojunction or both, leading to the band-edge emission of *n*-GaN and the YL band in the EL spectra. Figure 2(b) illustrates that a shoulder peak at approximately 368 nm showed the contention of band-edge emission of *n*-GaN. Reducing the thickness of the *n*-GaN layer in LED-I is a possible solution for decreasing the transit time of holes from the *p*-GaN layer of surface *p–n* junction to the QWs.

However, the thickness of the *n*-GaN layer in LED-I must be larger than the width of the depletion region, which is governed by the carrier concentrations of *p*- and *n*-GaN layers placed on the MQW structure. This inherent problem can be solved by placing the *p*-GaN layer between the *n*-GaN layer and QWs, but this scheme required dry etching for the exposure of the buried *p*-GaN layer associated with a suitable metallization process (e.g., Ni/Au) for the formation of anode electrodes when the selective-area regrowth technique is not performed. However, plasma damage originating from dry etching on the etched *p*-GaN surface often results in high-resistivity electrode contact and poor device performance^16–18^. Meanwhile, LED-II contained a regrown *n*-GaN layer on the *p*-GaN top layer of its InGaN/GaN MQW structure. The regrown layer formed a surface *p–n* homojunction (mesa) on the MQW structure after the removal of the SiO_2_ mask layer. In other words, the GaN *p–n* homojunction was formed without plasma damage on the *p*-GaN layer. In the fabrication of the devices, Cr/Au and ITO contacts were deposited on the regrown *n*-GaN and *p*-GaN layers, respectively. The Cr/Au metal contacts on the regrown *n*-GaN layer exhibited a typical specific contact resistance of approximately 1.1 × 10^−5^ Ω cm^2^, which was determined by a transfer length method. These results indicated that the electron concentration of the regrown *n*-GaN area was sufficiently high to result in ohmic contact via the tunneling mechanism of carrier transport^19,20^. The ITO contacts on the *p*-GaN layer exhibited a typical specific contact resistance of approximately 7.4 × 10^−3^ Ω cm^2^.

**Figure 3:**
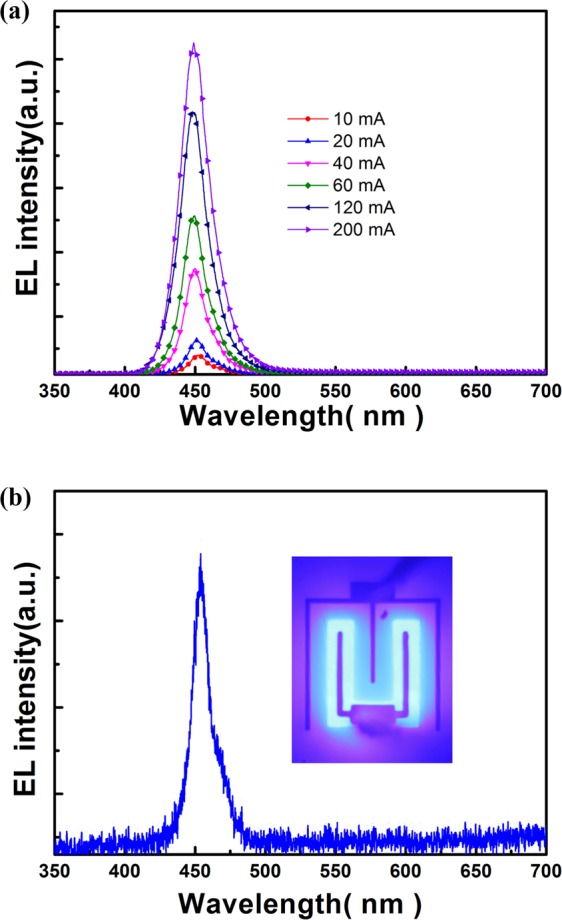
(**a**) Typical current-dependent EL spectra of LED-II; (**b**) typical EL spectrum of LED-II driven at a current of 5 mA. The inset of (**b**) displays the emission image of the fabricated LED-II.

**Figure 4:**
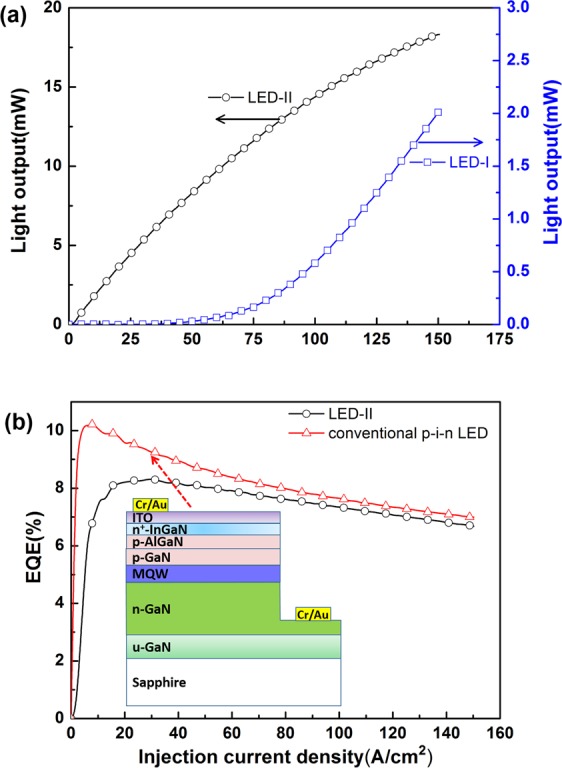
(**a**) Typical curves of light output versus the injection current of LED-I and LED-II; (**b**) normalized EQE as a function of injection current density for LED-II and conventional p-i-n LEDs. The inset of (**b**) displays a schematic layer structure of conventional p-i-n LED.

In LED-II, the p-GaN layer was placed between the n-GaN top layer and InGaN/GaN MQW active region. This scheme would be beneficial to the injection of holes into the MQW, compared with the LED-I. Figure 3(a) displays the current-dependent EL spectra of LED-II. Apart from the absence of YL peak in the EL spectra, reliable EL spectra can be obtained even when the driving current was as low as 5 mA, as shown in Fig. 3(b). In LED-II, the radiative recombination of injected carriers in the GaN *p–n* homojunction can be neglected owing to the insufficient band-edge emission of GaN at approximately 365 nm. In LED-II, the p–GaN layer was placed near the MQW active layer, which is beneficial to the hole injection in the QWs. Consequently, LED-II exhibited superior emission property compared with LED-I. In LED-I, the electrons directly diffuse from the n-GaN layer to the QWs. The part of holes first passes through the n-GaN layer of the GaN p–n junction above the QWs and then diffuse to the QWs before they recombine with electrons. Given that the diffusion length of holes is shorter than that of electrons in GaN, the hole injection efficiency in LED-I should be lower than that of LED-II especially at a low driving current. This difference can be indirectly confirmed by comparing the current-dependent EL spectra shown in Figs 2 and 3. As shown in the inset of Fig. 2(a), the emission image indicated significant current crowding at a driving current of 100 mA. Thus, the light output of LED-I was low. Figure 3(b) shows the typical EL spectrum of LED-II driven at a current of 5 mA. The inset of Fig. 3(b) displays the emission image of fabricated LED-II. The emission image indicated that the current (i.e., carrier) injection from the surface *p–n* GaN junction to QWs was laterally and uniformly spread. This result reflected the difference in light output power between the two types of LEDs. Figure 4(a) shows the curves of light output power versus the injection current (L-I curve) of LED-I and LED-II. In this study, the output power of LED-I was as low as the detection limit of our integrated sphere. Therefore, the light output of LED-I displayed in Fig. 4(a) was far lower than that of LED-II. The scheme of an n-GaN layer regrown on p-GaN facilitated the carrier injection to the QWs and current spreading, thus resulting in a considerable difference in light output between LED-I and LED-II. This condition can be tentatively clarified by examining the *I–V* characteristics of the fabricated LEDs.

As shown in Fig. 4(a), the L-I curve of LED-I exhibited a threshold at 50 mA because most of the injected carriers laterally moved within the surface *p–n* homojunction rather than perpendicularly diffusing into the buried QWs when the injection current was lower than 50 mA. Most of the injected carriers, especially for holes, were thus confined in the surface *p–n* junctions and recombined with the defect states. Therefore, the emission intensity was extremely low. Although the emission intensity increased as the injection current exceeded 80 mA, the light output of LED-I closed to the detection limit of our integrated sphere. However, the light output power of LED-II monotonically increased with injection current, and the reliable light output was obtained even when the injection current was below 5 mA. This result indicates that the current injection was more efficient in LED-II than in LED-I.

Figure 4(b) shows the EQE as a function of injection current density for LED-II and conventional p-i-n LEDs. The EQE of LED-II initially increased and slightly decreased with the further increase in injection currents. This efficiency droop trend is similar to that of conventional GaN-based blue LED^6^. For a conventional GaN/sapphire LED featuring a vertical *p–i–n* mesa, the applied electric field perpendicularly crossed the MQW active layer. Therefore, the electrons and holes diffused from the *n*-GaN and *p*-GaN layers, respectively, which were placed on the two sides of MQW active layer during forward operation, as shown in the inset of Fig. 4(b). In conventional LEDs, most of the radiative recombination events occur at the QW nearest to the *p*-layer^21^ because of the low diffusion length of the holes. The accumulation of the carriers amplifies the Auger recombination rate, resulting in efficiency droop^22–24^. As shown in Fig. 4(b), the typical EQE of conventional p-i-n LEDs sharply increased to a peak value when the current density increased to approximately 6.5 A/cm^2^ and started to droop with the further increase in current. This condition is a typical characteristic of an InGaN-based MQW p-i-n LED grown on c-faced sapphire. LED-II showed a typical trend in which EQE is a function of injection current density, As shown in Fig. 4(b), and this trend was slightly different from that observed in conventional p-i-n LEDs. The EQE rapidly increased when the current densities were less than 20 A/cm^2^ and reached a peak at a current density of approximately 30 A/cm^2^. The efficiency droop percentage in LED-II was lower than that of conventional p-i-n LEDs. The efficiency droop ratio, which is defined as the peak EQE to the EQE at a current density of 150 A/cm^2^ (i.e., ${{\rm{EQE}}}_{{\rm{peak}}}-{{\rm{EQE}}}_{200{\rm{A}}/{{\rm{cm}}}^{2}}/{{\rm{EQE}}}_{{\rm{peak}}}$), was alleviated from 30% in conventional p-i-n LEDs to 20% in LED-II. For LED-II, the external electric field was laterally applied to the surface *p–n* junction surface above the MQW active layer rather than on the MQW active layer during the forward operation. Thus, the electrons and holes diffused from the surface *p–n* junction placed on one side of the MQW active layer.

**Figure 5:**
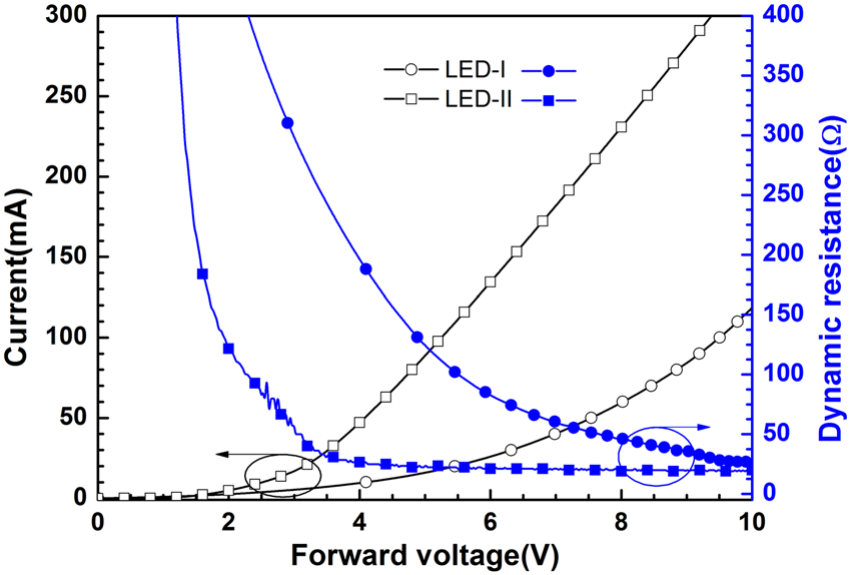
Typical current–voltage–resistance (*I–V–R*) characteristics obtained from the fabricated LED-I and LED-II.

Figure 5 displays the typical *I–V* characteristics obtained from the fabricated LED-I and LED-II. The increase in *V*_t_ of LED-I was attributed to poor current spreading, which resulted in high series resistance (*R*_s_). Regarding the dynamic resistance (*R*_d_), LED-II exhibited a typical characteristic comparable to those of conventional GaN-based LEDs. In general, *R*_d_ sharply decreases and is saturated at a fixed value when the forward voltage markedly exceeds the *V*_t_. Saturated *R*_d_ or *R*_s_ was generally lower than 20 Ω in a typical GaN-based LED with a chip size of 350 × 350 μm^2^ ^2^. In this study, the saturated *R*_d_ values were 26 and 20 Ω for LED-I and LED-II, respectively. The relatively high *R*_s_ was due to the inferior material quality of the regrown *p*-GaN layer in LED-I relative to that of the as-grown *p*-GaN layer in LED-II; this poor material quality resulted in poor current spreading. The difference in electrical characteristics between LED-I and LED-II was also reflected on the L-I characteristics (Fig. 4(a)). Although the *R*_s_ of the recently reported GaN-based p-i-n LED was smaller than our devices, which were fabricated by a nonoptimal process and electrode design, the relative lower *R*_s_ obtained from the reported LEDs may be due to the large areas and different electrode configurations (e.g., flip-chip or vertical type) of the devices^25^.

## Conclusion

We presented GaN-based LED structures with surface GaN *p–n* junctions, which served as carrier injectors, formed through selective area regrowth on their respective InGaN/GaN MQW structures. During the forward operation, external fields were directly applied to the surface *p–n* junction carrier injector rather than to the MQW active layer. The experimental results indicated that the LED structure equipped with a *p*-GaN layer placed between MQW and the regrown *n*-GaN top layer exhibited higher light output power and better current spreading than those of LEDs with regrown *p*-GaN top layer. This result was attributed to the difference in hole injection efficiency between the two structures.

## Supplementary information

Light-emitting diodes with surface gallium nitride p–n homojunction structure formed by selective area regrowth
