# Supplementary material for: Light-emitting diodes with surface gallium nitride p–n homojunction structure formed by selective area regrowth
Source: Sci Rep. 2019 Mar 1;9:3243. doi: 10.1038/s41598-019-40095-7 (PMC6397187; doi:10.1038/s41598-019-40095-7)
Supplement: Supplementary file 1 — Light-emitting diodes with surface gallium nitride p–n homojunction structure formed by selective area regrowth [file 41598_2019_40095_MOESM1_ESM.docx]

**Light-emitting diodes with surface gallium nitride *p–n* homojunction structure formed by selective area regrowth**

Ming-Lun Lee,^1^ Shih-Sian Wang,^2^ Yu-Hsiang Yeh,^1,2^ [Po-Hsun Liao](http://ndltd.ncl.edu.tw/cgi-bin/gs32/gsweb.cgi/ccd=tdrHFV/search?q=aue=%22Po-Hsun%20Liao%22.&searchmode=basic),^2^ and Jinn-Kong Sheu,^2,*^

^1^ Department of Electro-Optical Engineering, Southern Taiwan University of Science and Technology, Tainan 71001, Taiwan

^2^ Department of Photonics and Advanced Optoelectronic Technology Center, National Cheng Kung University, Tainan City 70101, Taiwan

*Contact Email: [jksheu@mail.ncku.edu.tw](mailto:jksheu@mail.ncku.edu.tw) (J. K. Sheu)

**Supporting information:**

Figure S1 depicts the schematic conventional p-i-n GaN structure. The conventional p-i-n GaN LED wafers were all grown on c-face sapphire substrates by metal-organic vapor-phase epitaxy (MOVPE) system. Trimethylgallium, trimethylindium, and ammonia were used as the sources for gallium, indium, and nitrogen, respectively, to grow the p-i-n GaN LED wafers. The layer structure of the p-i-n GaN LED wafer consisted consisted of a 30 nm-thick GaN nucleation layer and a 1 μm-thick u-GaN layer grown at 530 °C and 1000°C, respectively. Subsequently, a 2 µm-thick Si-doped n-GaN layers with electron concentration of ~3x10^18^/cm^3^, which used SiH_4_ as doping source. Next, a undoped In_0.23_Ga_0.77_N/GaN MQW(multi-quantum well, 10 pairs) structure grown at 750 °C followed by a 80-nm-thick Mg-doped p-Al_0.15_Ga_0.85_N electron blocking layer (EBL) and a 0.2 μm-thick Mg-doped p-GaN contact layer grown at 950 °C. It should be noted that the CP_2_Mg flow rate was 0.2 μmole/min for the growth of p-Al_0.15_Ga_0.85_N and p-GaN layers, respectively. On the other hand, the growth pressure was 100 torr for the growth of p-Al_0.15_Ga_0.85_N and p-GaN layers. As a result, the p-Al_0.15_Ga_0.85_N and p-GaN layers exhibited hole concentration of 1×10^17^ cm^-3^ and 3×10^17^ cm^-3^, respectively, which were determined by Hall-effect measurement. Afterwards, a heavily Si-doped In_0.3_Ga_0.7_N layer with thickness of around 3 nm was grown at 750°C on the p-GaN to form the tunneling junction. The 3 nm-thick In_0.3_Ga_0.7_N layer exhibited electron concentration of ~1×10^20^ cm^-3^. For the fabrication of LED chips, a 300nm-thick indium tin oxide (ITO) layer was deposited on the In_0.3_Ga_0.7_N top layer as a transparent contact layer (TCL). Then, plasma etching using chlorine-based gas was performed to expose the n-type GaN underlying layer. Finally, Cr/Au-based metal layers were deposited onto the exposed n-type layer and the ITO layer to serve as the n-type and p-type electrodes, respectively^1-3^.

**
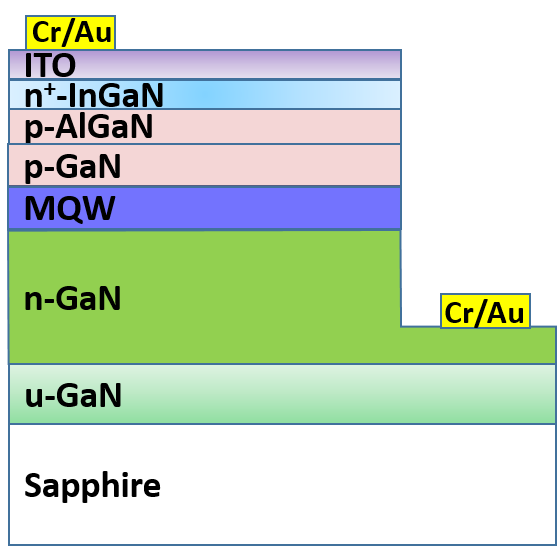
**

**Figure S1.** A schematic layer structure of conventional p-i-n LED.

**
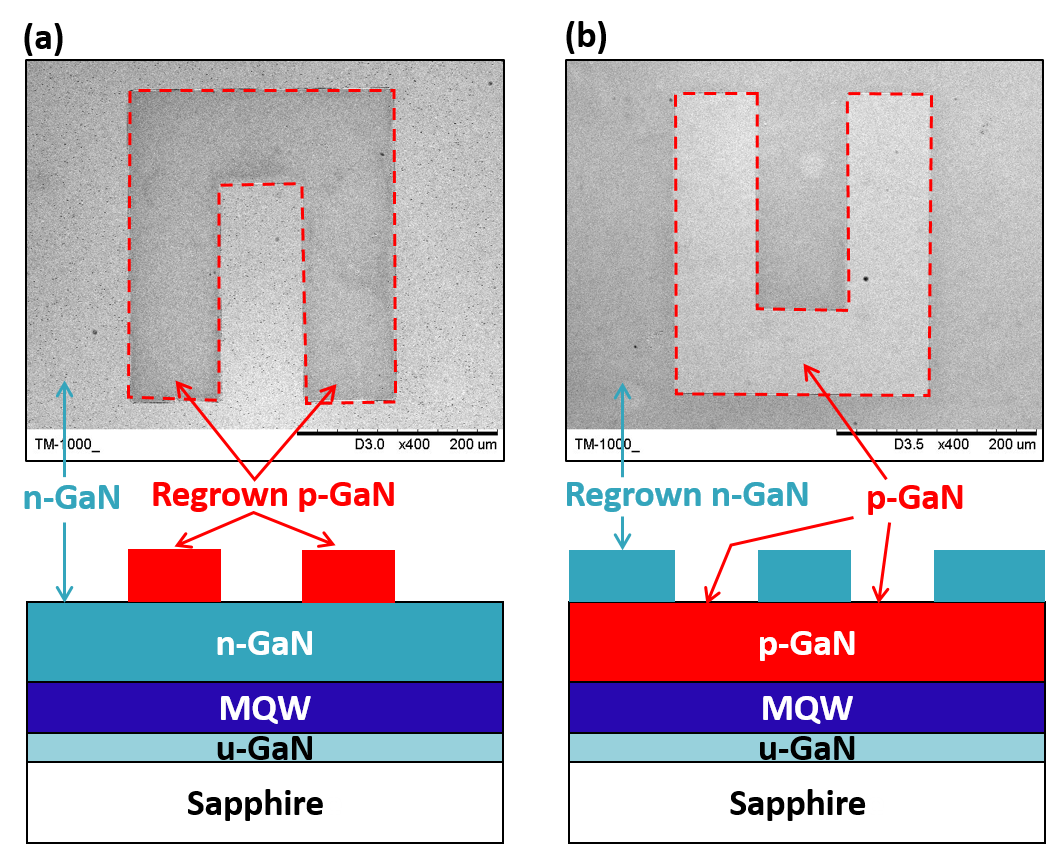
**

**Figure S2.** (a) typical SEM image taken from the samples with regrown p-GaN mesa on the n-GaN/MQW template (b) typical SEM image taken from the samples with regrown n-GaN mesa on the p-GaN/MQW template.

All the epitaxial regrowth processes were conducted by MOVPE reactor ( Emcore D-180 ). Schematic processing steps including the formation of SiO_2_ mask layer were described in the main text and Fig.1. Figure S2(a) shows the schematics that a p-GaN top layer was regrown on the templates with an n-GaN cap layer. In this study, there is no specific thermal baking procedure for the regrowth process. The regrowth procedures started from a ramp of susceptor temperature from room temperature to 950 °C within 20 minutes. The H_2_ and NH_3_ with flow rates of 10 and 8 liters/min, respectively were introduced into the reactor during the temperature ramp. Trimethylgallium (TMGa), biscyclopentadienemagnesium (CP_2_Mg) and NH_3_ were used as the sources for gallium, magnesium, and nitrogen, respectively. At a reactor pressure of 100 torr, the flow rates of TMGa, and CP_2_Mg were 157 and 0.2 μmole/min, respectively, during the regrowth of the p-GaN layer. Based on such a growth condition, the regrown p-GaN layer exhibited a hole concentration of approximately of 3×10^17^cm^-3^. As shown in Fig S2(b), an n-GaN top layer was regrown on the templates with a p-GaN cap layer. All the regrowth parameters of the n-GaN were the same as the regrowth of p-GaN top layer except for the doping source(i.e., SiH_4_). The flow rate of the SiH_4_ was 0.05 μmole/min during the regrowth of n-GaN layer. Based on such a growth condition, the regrown n-GaN layer exhibited an electron concentration of approximately of 3×10^18^ cm^-3^. The carrier concentrations of the regrown n-GaN and p-GaN layers were indirectly determined by Hall-effect measurement using epitaxial wafers regrown on templates without SiO_2_ mask layer. In other words, the epitaxial wafers without SiO_2_ mask layer were regrown at the same time during the selective regrowth of p-GaN or n-GaN layers.


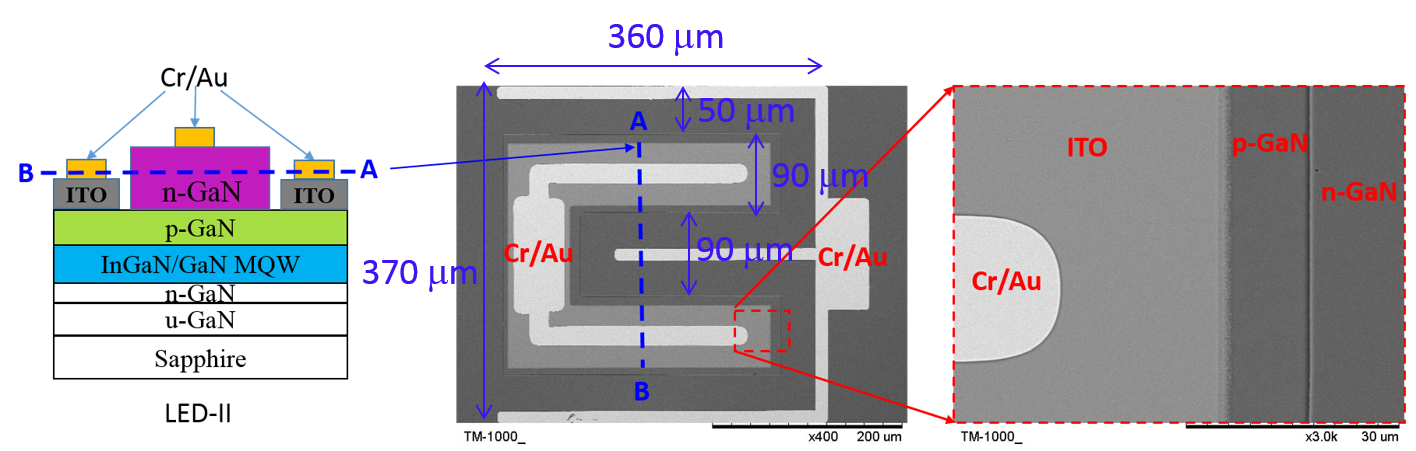


**Figure S3.** Layer structure of LED-II and typical SEM image of the fabricated LED-II.


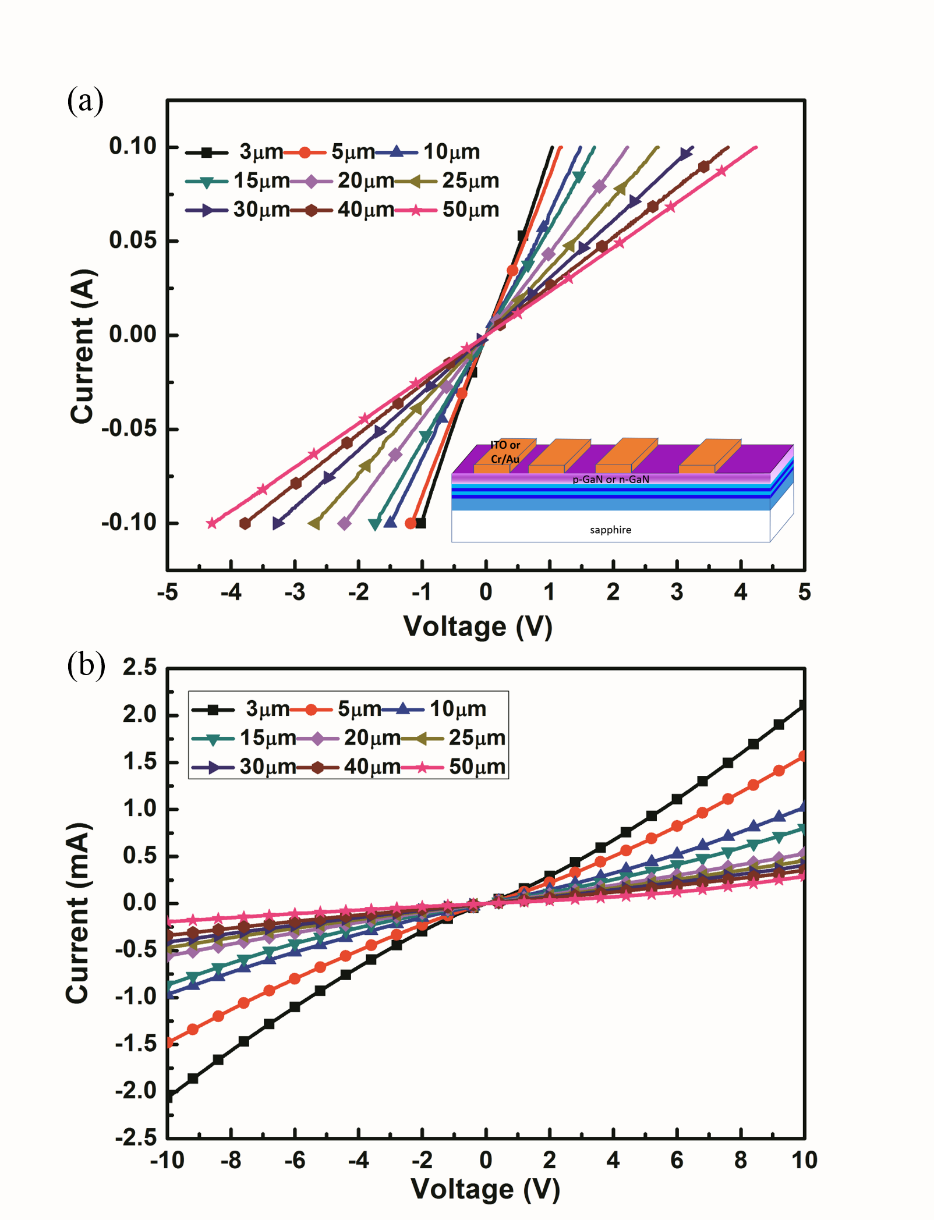


**Figure S4.** Typical *I–V* characteristics of (a) Cr/Au contacts on the regrown *n*-GaN layer (b) ITO contacts on the as-grown *p*-GaN layer.

Figures S4 (a) and (b) display the *I–V* characteristics of Cr/Au and ITO contacts on the regrown *n*-GaN and p-GaN layers, respectively. The data were measured from two adjacent transfer length method (TLM) metal pads with different spacings, as shown in the inset of Fig. S4(a). Cr/Au(50 /250 nm) metal contacts on the regrown *n*-GaN exhibited linear *I–V* characteristics that corresponded to a specific contact resistance of 1.1×10^−5^ Ω cm^2^. Such results indicated that the electron concentration of regrown *n*-GaN area was sufficiently high to result in ohmic contact via the tunneling mechanism of carrier transport^1^. By contrast, the ITO contacts with TLM patterns on the p-GaN layer exhibited a slightly nonlinear characteristic corresponding to a specific contact resistance of 7.4×10^−3^ Ω cm^2^. The slightly high contact resistance obtained from our samples was due to the Si atoms diffused in *p*-GaN from the SiO_2_ cap layer that function as donors during regrowth; the atoms thereby reduced the hole concentration of the *p*-GaN surface layer. In addition, the SiO_2_ capped *p*-GaN area possessed increased surface defect density compared with the as-grown *p*-GaN because the Si atoms possibly reacted with Ga atoms of the GaN layer to from the Ga silicide at the *p*-GaN surface during regrowth. This process resulted in an adverse effect on carrier transport at ITO/p-GaN interface and increased contact resistance.


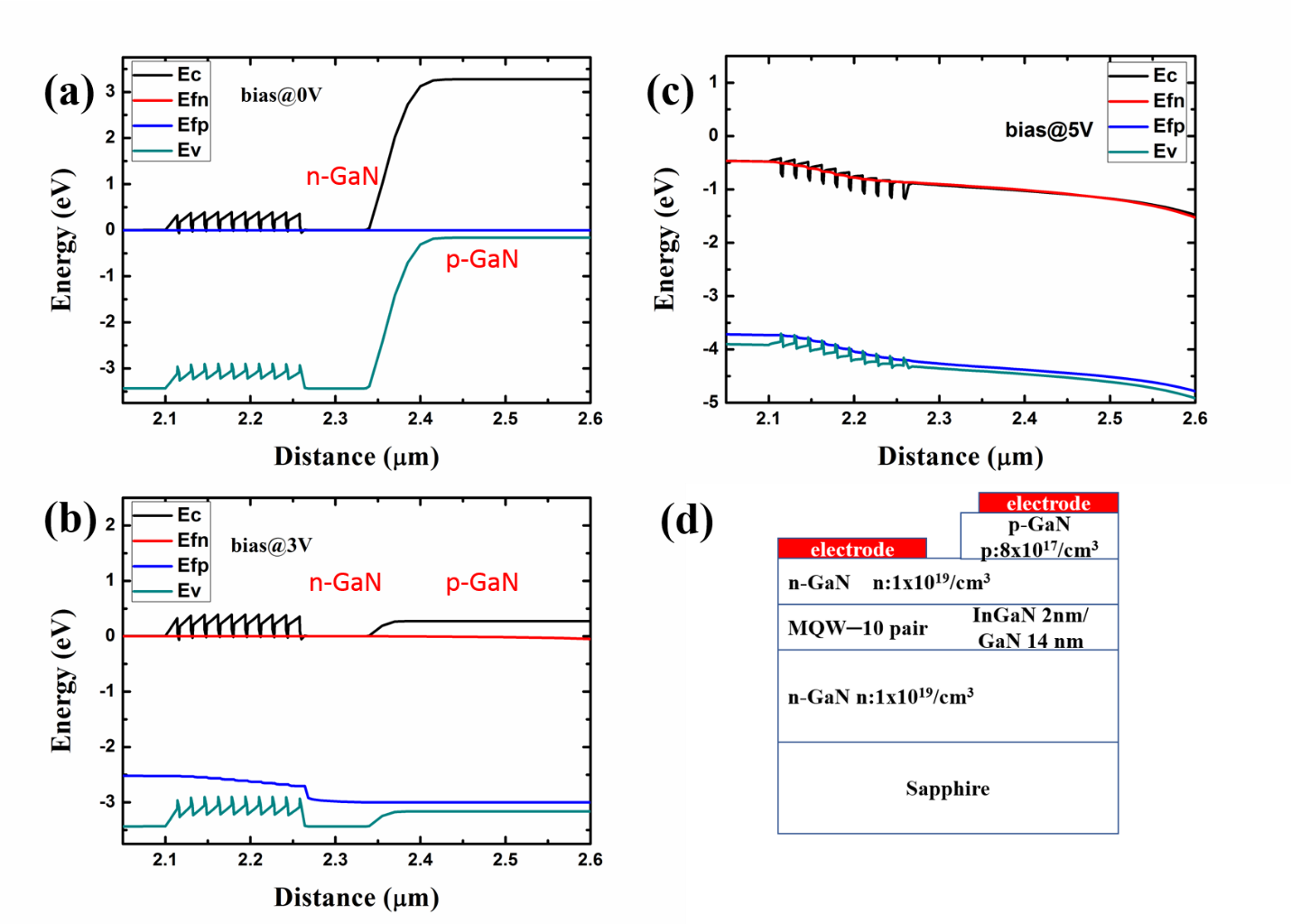


**Figure S5**. Simulation results of band diagrams for LED-I with regrown p-GaN mesa on the n-GaN/MQW template (a)biased at 0 V(b) biased at 3 V(c) biased at 5V (d) schematic layer structure LED-I for the simulation.


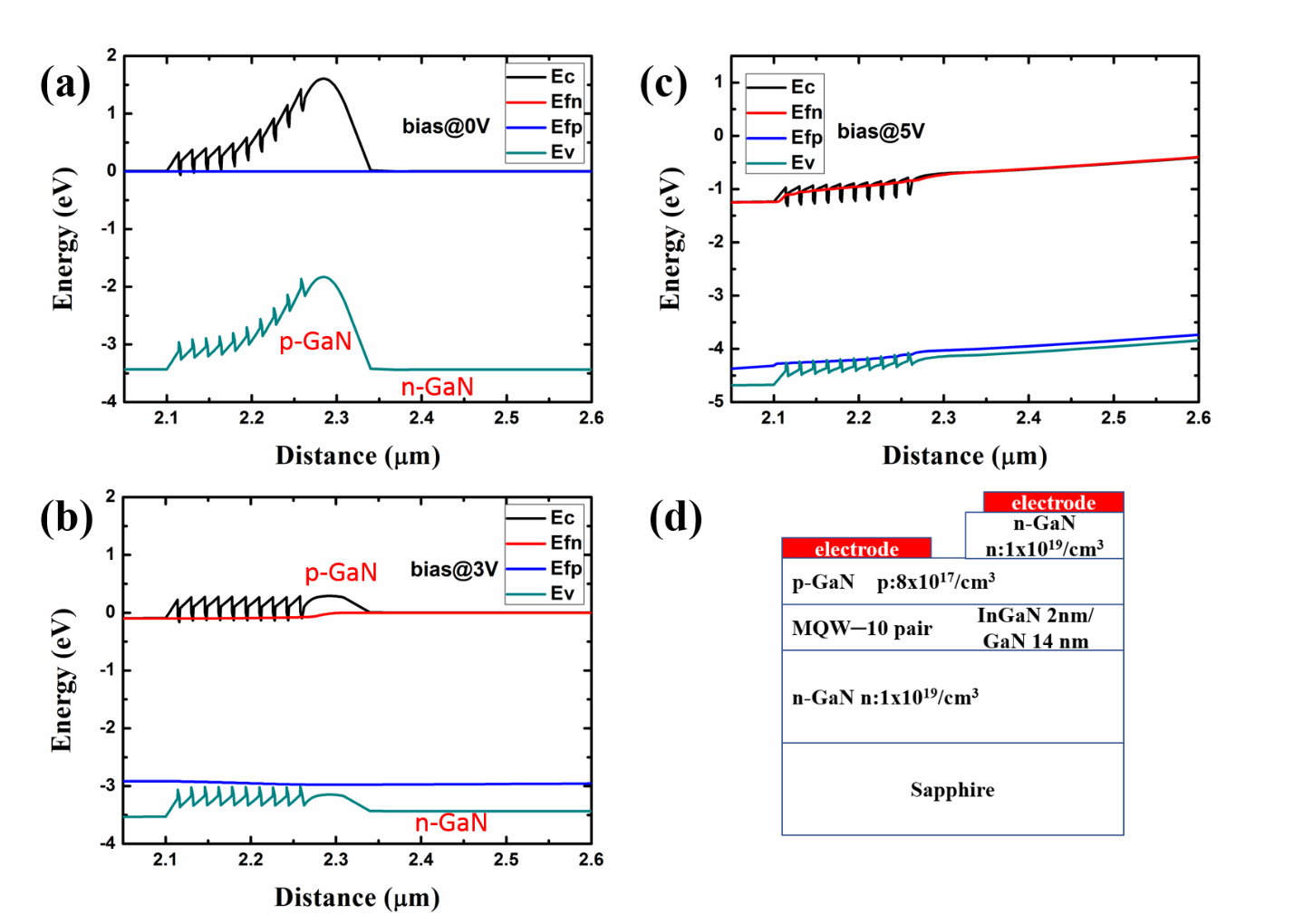


Figure S6. Simulation results of band diagrams for LED-II with regrown n-GaN mesa on the p-GaN/MQW template (a)biased at 0 V(b) biased at 3 V(c) biased at 5 V (d) schematic layer structure LED-II for the simulation.

# The band diagrams were performed using a fully 2-D self-consistent finite-element method (FEM) solver to solve Poisson and drift–diffusion equations self-consistently^4^. The simulation program can be downloaded at <http://yrwu-wk.ee.ntu.edu.tw/>.

# References

# 1. Lee, M. L., Sheu, J. K., & Hu, C. C. Non-alloyed Cr/Au-based Ohmic contacts to n-GaN. Appl. Phys. Lett. 91, 182106 (2007).

2. Sheu, J. K., Tsai, J. M., Shei, S. C., Lai, W. C., Wen, T. C., Kou, C. H., Su, Y. K., Chang, S. J., & Chi, G. C. Low-operation voltage of InGaN/GaN light-emitting diodes with Si-doped In_0.23_Ga_0.77_N/GaN short-period superlattice tunneling contact layer. IEEE Electron Device Lett. 22, 460-462 (2001).

3. Chang, C.S., Chang, S. J., Su, Y.K., Kuo, C.H., Lai, W.C., Lin, Y.C., Hsu, Y.P., Shei, S.C., Tsai, J.M., Lo, H.M., Ke, J.C., & Sheu, J.K. High brightness InGaN green LEDs with an ITO on n^++^-SPS upper contact. IEEE Tran. Electron Devices. 50, 2208-2212 (2003).

4. Li, C.K. & Wu, Y.R. Study on the current spreading effect and light extraction enhancement of vertical GaN/InGaN LEDs. [IEEE Tran. Electron Devices](https://ieeexplore.ieee.org/xpl/RecentIssue.jsp?punumber=16). 59, 400-407 (2012).
